# Mass Fingerprinting of the Venom and Transcriptome of Venom Gland of Scorpion *Centruroides tecomanus*

**DOI:** 10.1371/journal.pone.0066486

**Published:** 2013-06-20

**Authors:** Laura L. Valdez-Velázquez, Verónica Quintero-Hernández, Maria Teresa Romero-Gutiérrez, Fredy I. V. Coronas, Lourival D. Possani

**Affiliations:** 1 Facultad de Ciencias Químicas and Facultad de Medicina, Universidad de Colima, Colima, México; 2 Instituto de Biotecnología, Universidad Nacional Autónoma de México, Cuernavaca, Morelos, México; National Central University, Taiwan

## Abstract

*Centruroides tecomanus* is a Mexican scorpion endemic of the State of Colima, that causes human fatalities. This communication describes a proteome analysis obtained from milked venom and a transcriptome analysis from a cDNA library constructed from two pairs of venom glands of this scorpion. High perfomance liquid chromatography separation of soluble venom produced 80 fractions, from which at least 104 individual components were identified by mass spectrometry analysis, showing to contain molecular masses from 259 to 44,392 Da. Most of these components are within the expected molecular masses for Na^+^- and K^+^-channel specific toxic peptides, supporting the clinical findings of intoxication, when humans are stung by this scorpion. From the cDNA library 162 clones were randomly chosen, from which 130 sequences of good quality were identified and were clustered in 28 contigs containing, each, two or more expressed sequence tags (EST) and 49 singlets with only one EST. Deduced amino acid sequence analysis from 53% of the total ESTs showed that 81% (24 sequences) are similar to known toxic peptides that affect Na^+^-channel activity, and 19% (7 unique sequences) are similar to K^+^-channel especific toxins. Out of the 31 sequences, at least 8 peptides were confirmed by direct Edman degradation, using components isolated directly from the venom. The remaining 19%, 4%, 4%, 15% and 5% of the ESTs correspond respectively to proteins involved in cellular processes, antimicrobial peptides, venom components, proteins without defined function and sequences without similarity in databases. Among the cloned genes are those similar to metalloproteinases.

## Introduction

Scorpionism is the epidemiologic word commonly used to define human accidents caused by scorpion stings, which occurs in many parts of the world, but specifically in seven different geographical regions: three in Africa (North, Sahelian region and South), Asia Minor, South of India, Mexico and South America, where circa 2.3 billions of human persons are at risk [1].

There are approximately 1,500 different species of scorpion described in the world, from which over 200 are found in Mexico [2]. Colima is a Mexican state on the West coast, which due to the high diversity of climate and geographical variations, hosts scorpions species belonging to four different families [3]. Within the family Buthidae, the genus Centruroides is the most important one, because it contains venomous species to humans and includes the most toxic species of the world [4], [5].

Earlier taxonomic classification [4] of this scorpion placed this species within the family Buthidae, genus *Centruroides,* species *limpidus,* sub-especies *tecomanus*. Under this denomination (*Centruroides limpidus tecomanus*), a few articles related to venom components [6]–[8] were published by our group, as it will be shortly described below. Recently, Ponce-Saavedra et al [9] renamed it, as a *bona fide* species (*Centruroides tecomanus*), here thereafter abbreviated *C. tecomanus*. It plays an important health problem in the country, especially in the State of Colima [10].

Scorpion venoms are a complex mixture of peptides and proteins with a broad range of biological activities, among which are peptides that impair proper functioning of ion-channels present in excitable and non-excitable membranes, mainly Na^+^- and K^+^-ion channels (reviewed in [11]), [12]–[17], but also Ca^2+^-channels [18]–[22]. A chloride channel (or transporter) specific peptide was also described [23]. The toxins that recognize sodium channels usually modify the gating mechanism of the channel. They are modulators of the function, either prolonging the kinetics of closing time (alpha-scorpion toxin, abbreviated α-NaScTx) or openning the channels at less depolarized potentials (beta-scorpion toxin, abbreviated β-NaScTx) [24]–[27]. However, K^+^-channel specific scorpion peptides are usually blockers of the channels and were divided in α-, β-, γ- and κ-channel toxins [12]. Many other components have been found and described to occur in scorpion venoms (reviewed in [17]).

Concerning the venom of *C. tecomanus* the only biochemical data available indicates the presence of a few toxic fractions II.9.3 [6], II.22.5 and II.21.4 [8] for which the N-terminal sequence were determined. In addition a complete primary structure of toxin Clt1, a peptide with 66 amino acid residues that affect neuronal Na^+^-channels was described [7]. The earlier strategy used for separation of these components was gel filtration on Sephadex G-50 columns, followed by ion-exchange chromatography. The chemical characterization was done by automatic Edman degradation [6]–[8]. Latter, the isolation and identification of scorpion venom components from other species, were performed by means of high performance liquid chromatography (HPLC), followed by Edman degradation or mass spectrometry analysis [28]–[30]. More recently, the identification of venom components are performed by constructing cDNA libraries of venom glands obtained from scorpions of different families [31]–[44].

The results obtained by this more recent methodology, using cDNA libraries, indicate the presence of a rich biodiversity and variability of components in the species studied. Apart from the peptides that modify ion-channel permeability, initially isolated and characterized by classical biochemical methods, many other components were placed in evidence, such as: factors that active lipolysis, phospholipase A2, serine-proteases, metalloproteinases, protein homologs of tick salivary glands, precursors of cytolytic peptides, proteins rich in cysteine contents and a great number of proteins and peptides deduced from the ESTs for which the function is still unknown [45], [46] (reviewed in [47]). The results of cDNA analysis also shows that the venom glands of scorpions have many components related to cellular processes, protein synthesis, protein trafficking and others [31]–[38].

In this communication we report results of a transcriptome analysis obtained from a cDNA library prepared from the venom glands of two *C. tecomanus* scorpions, as well as a proteomic analysis from the soluble venom of this species. Eight peptides assumed to exist, based on the genes obtained from the cDNA library, were actually purified from the venom and their amino acid sequence were confirmed by direct amino acid sequencing, using Edman degradation and mass spectrometry. Seven are peptides assumed to affect Na^+^-channels, whereas one is specific from K^+^-channels.

In our opinion this is relevant information, since Mexico is one of the seven areas in the world, where scorpionism is important, and the State of Colima has one of the most dangerous scorpions to humans.

## Materials and Methods

### Biological Materials and Venom Extraction

The collection of scorpions of the species *C. tecomanus* took place in the community of Coquimatlán, state of Colima, Mexico (latitude 19°13′57.19′ ´N; longitude 103°49′46.05′ ´O; elevation 365 m over the sea level, under official permit of the Federal Government by *Secretaria de Medio Ambiente y Recursos Naturales* (reference: SGPA/DGVS/10638/11 of Dic/03/2011). The specimens were identified and classified using taxonomic keys prepared by [4], [9], [48], [49]. Prior manipulations the animals were kept in captivity (standard conditions of temperature, light and dark periods, water *ad libitum*) for 15 days. The extraction of venom was conducted using 27 scorpions, submitted to electrical stimulation (15 Volts shock applied to the animals). The venom was solubilized in 0.4 ml distilled water and was centrifuged at 14000 rpm for 15 min. The supernatant was removed, lyophilized and kept at −20°C until use.

### Chromatographic Separation of Soluble Venom

The content of venom protein was determined by optical density assuming that one unit of absorbance at 280 nm, in a 1 cm cuvette pathway, is equivalent to 1 mg/ml concentration. A total of 2.3 mg protein of soluble venom was separated by high performance liquid chromatography (HPLC) on a reverse phase analytical column C18 (dimensions of 250×10 mm) obtained from Vydac (Hisperia, CA, USA). The components were purified using a linear gradient of solvent A (0.12% trifluoroacetic acid –TFA- in water) to 60%, solvent B (0.10% TFA in acetonitrile) for 60 minutes with a flow rate of 1 ml/min. The fractions were collected manually by monitoring the absorbance at 230 nm and then dried in a Savant Speed Vac SC210A apparatus (Albertville, MN, USA).

### Mass Spectrometry Analysis

The various fractions obtained from the HPLC separation were dissolved in 50% acetonitrile containing 1% acetic acid to a final concentration of *circa* 0.1 to 0.5 mg/ml. This concentration was estimated based on the area under the curve of the various sub-fractions as indicated by the integral obtained from the chromatogram of the HPLC separation. All samples were analyzed using a LCQ Fleet mass spectrometer (Thermo Finnigan, San Jose, CA, USA). A few components with high molecular weight were identified using a Hybrid Orbitrap-Veloz mass spectrometer from the same company with nano-electro spray ionizations source. Fractions were reconstituted in 50% acetonitrile with 1% acetic acid and submitted to the mass spectrometer. Automatic and manual deconvolutions were performed to determine the average molecular mass composition of the components. The automatic deconvolutions were performed by X-tract tool, part of the Xcalibur software.

### cDNA Library Construction

A cDNA library was constructed from total RNA extracted from the scorpion *C. tecomanus*. Each scorpion in the last postabdominal segment (telson) has a pair of venom glands, and two specimens (four glands) were used for homogenization of tissue. The scorpions were milked 5 days before RNA extraction. For RNA isolation the ‘Total RNA Isolation System’ of Promega (Madison,WI) was used. With this material a full-length cDNA library was prepared using the Creator™ SMART™ cDNA Library Construction Kit (CLONTECH Lab., Palo Alto,CA). For the first-strand cDNA synthesis, the oligonucleotides of the kit: SMART IV™ Oligonucleotide (5′-AAGCAGTGGTATCAACGCAGAGTGGCCATTACGGCCGGG-3′) and CDSIII/3′ PCR Primer (5′-ATTCTAGAGGCCGAGGCGGCCGACATG-d(T)_30_N_−1_N-3′) were used as primers. For cDNA amplification, the oligonucleotides 5′ PCR Primer (5′-AAGCAGTGGTATCAACGCAGAGT-3′) and CDSIII/3′ PCR Primer were used. The conditions used for both PCRs were according to the provideŕs instructions.

The double-stranded cDNA contained *Sfi*I A and B restriction sites on the cDNA ends, was digested with *Sfi*I restriction enzyme and fractionated on a CHROMA SPIN-400 column according to molecular size (Creator™ SMART™ cDNA Library Construction Kit). Fractions containing cDNA of the desired size were pooled and concentrated by ethanol precipitation. The purified cDNA was ligated to the *Sfi*I sites of the pDNR-LIB plasmid (Creator™ SMART™ cDNA Library Construction Kit) and the ligation reaction was used to transform *Escherichia coli* DH5 cells by electro-transformation. The titer of the non-amplified cDNA library obtained is in the order of 1×10^6^ cfu/mL, with 99% recombinant clones.

### DNA Sequencing and Bioinformatic Analyses

Plasmids of selected colonies were isolated according to a Standard alkaline lyses protocol, and single-pass sequencing of the 5′-termini was conducted with the primer T7 (5′-GTAATACGACTCACTATAGGG-3′) using an automatic machine (Model 3100, Applied Biosystems, Foster city, CA) according to the manufacturer’s instructions.

The nucleotide sequences obtained in this work are deposited in GenBank (EST database: dbEST JZ122265 - JZ122341). To extract the high quality sequence region, the ESTs were subjected to the Phred program [50] with the window length set to 100 and the standard quality to 20. The CrossMatch program was used to remove vector and *Escherichia coli* DNA sequences. ESTs that share an identity of >95 out of 100 nucleotides were assembled in contiguous sequences with the CAP3 program (http://pbil.univ-lyon1.fr/cap3.php), All these bioinformatic analysis were simultaneously performed at the http://www.biomol.unb.br/site of **“**Laboratório de Biologia Molecular – Universidade de Brasilia”**,** using default setup. *C. tecomanus* venom gland ESTs (clusters and singlets) were searched against nr database using blastx and blastn algorithms (http://www.ncbi.nlm.nih.gov/blast) with an e-value cutoff set to <10^−5^ to identify putative functions of the new ESTs. Additional search was performed with ORF Finder (Open Reading Frame Finder; http://www.ncbi.nlm.nih.gov/projects/gorf/), PROSITE (http://prosite.expasy.org/) and Pfam (http://pfam.sanger.ac.uk). The signal peptide was predicted with the SignalP 3.0 program (http://www.cbs.dtu.dk/services/SignalP/)., and prediction of propetide was performed by using Prop 1.0 Server (http://www.cbs.dtu.dk/services/ProP/). The theoretical molecular weights of the cDNA sequences were calculated using the program ProtParam (http://web.expasy.org/protparam). Multiple sequence alignments were done with the clustalw2 program (http://www.ebi.ac.uk/Tools/msa/clustalw2/) and the percentage of sequence identity were determined using the DNA Strider™ 1.3 program.

### Amino Acid Sequencing of Venom Components

Amino acid sequence determination of the N-terminal segment of the eight purified peptides was obtained by automatic Edman degradation into a Protein sequencer PPSQ-31A SHIMADZU (Kyoto, Japan), using the chemicals and procedures recommended by the provider.

## Results

### Separation of Venom Components and Mass Fingerprinting

**Figure 1:**
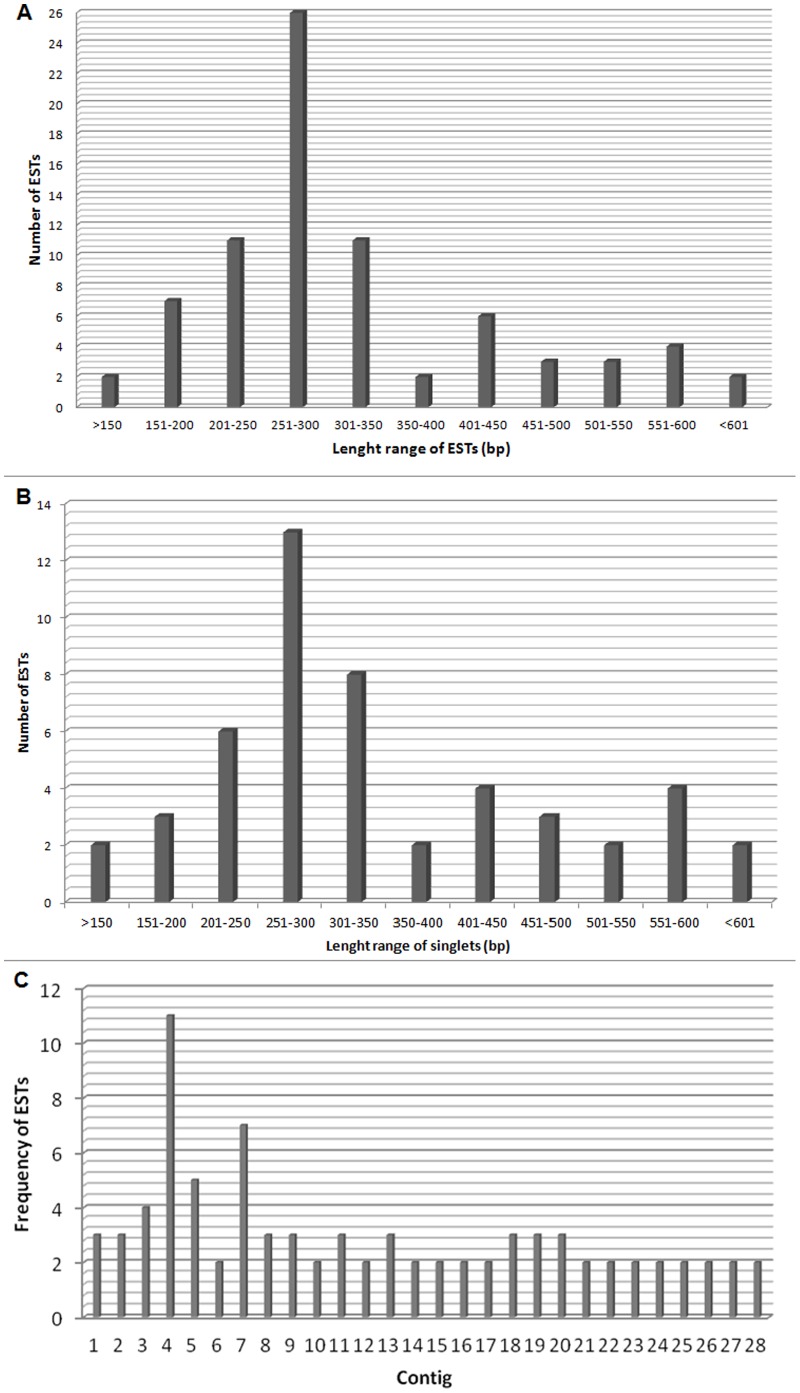
HPLC separation of soluble venom. Venom (2.3 mg) from *C. tecomanus* was separated in a C18 reverse-phase column using a linear gradient from solution A (water in 0.12% TFA) to 60% solution B (acetonitrile in 0.10% TFA) over 60 min.

**Figure 2:**
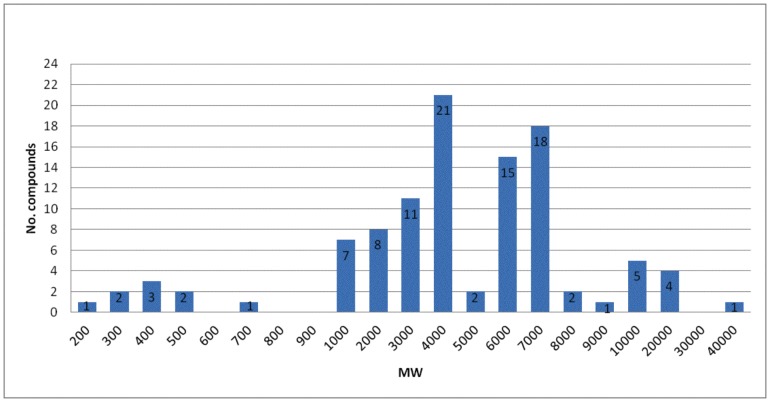
Molecular mass distribution of separated venom. The histogram shows the distribution of the 104 different molecular weights (MW), in Da, present in the venom of *C. tecomanus*. Peptides and proteins having MWs from 3.0, 4.0, 6.0 and 7.0 K Da are the most abundant components. The molecular weigths were obtained by LCQ Fleet and LTQ-Orbitrap-Veloz mass spectrometer (see materials and methods).

Separation of soluble venom by HPLC (Figure 1) shows more than 60 clear chromatographic peaks, which were collected in 80 distinct fractions. From these, at least 104 different components were identified by mass spectrometry determination, with molecular weights varying from 259 to 44, 392 Da. The results obtained are shown in Table 1. In a few cases, due to the overlapping of components in the various adjacent chromatographic peaks, the same peptide with the same molecular mass (or within one unit mass difference, which is the resolution of our LCQ apparatus) were found, and are listed in Table 1 by their retention time on the column. A few components (total 16) were not identified, either due to their chemical compositions or complexity (several components in the fractions, impeding individual bona fide identification). In figure 2 the molecular mass distribution of the venom components found in the 80 fractions are reported, clustered within different intervals of molecular weights, mostly 1000 Da apart from each other. Four groups of components with distinct molecular masses were found : 200–1000 Da (8.7%), 1000–5500 Da (47%), 5500–8500 Da (33.7%) and 8500–44392 Da (10.6%). The majority of peptides have a molecular weight between 3000 to 4000 and from 6000 to 7000, which usually correspond to peptides known to affect K^+^-channels and Na^+^-channels, respectively.

**Table 1:** Determination of molecular weights (MW) of the venom components from *C*. *tecomanus*, obtained by mass spectrometry.

RT (min)	MW (Da)	RT (min)	MW (Da)
**3.67**	ND	**29.91**	8302
**4.53**	486, 347	**30.30**	4105, 4210, 4361
**5.04**	ND	**30.48**	4105, 8212
**7.39**	ND	**30.92**	7014, 7292 (**Ct16**)
**7.63**	5361	**31.21**	7292 (**Ct16**)
**8.84**	449	**32.31**	7015, 4606, 9213
**12.06**	353	**32.93**	7427 (**Ct17**), 4605, 7378
**13.16**	259	**33.38**	7171, 7485
**13.63**	452	**33.51**	7171, 7347, 5164
**14.28**	ND	**34.05**	6333 (**Ct13**)
**15.63**	ND	**34.26**	6333 (**Ct13**)
**16.21**	ND	**35.19**	6335, 7591 (**Ct1a**)
**16.59**	ND	**35.92**	6335, 7591 (**Ct1a**)
**16.83**	1702	**36.32**	7091, 7613
**17.06**	568	**36.72**	7613
**17.56**	1918, 547.5	**37.03**	6989, 6839, 7612, 7288 (**Ct6**)
**17.71**	1918, 547.5	**37.69**	7099, 7609, 6989, 3744
**18.71**	2518, 2535	**37.99**	6992, 6858, 7271
**19.00**	2611	**38.29**	6858
**19.93**	1021, 2611	**38.65**	6858, 6817 (**Ct7**)
**20.51**	3806	**39.61**	3696,4715,6604,6814 (**Ct25**),13726
**20.94**	3807	**40.21**	4599, 4644, 4715, 6135, 6604, 6813(**Ct25**), 7012
**21.14**	3807, 4255 (Ct28)	**41.29**	6591
**21.92**	3807, 3679, 3552	**41.76**	6605, 6591
**22.50**	3804	**42.10**	6119, 6604
**23.21**	3420, 764	**44.37**	44392
**23.59**	3420, 1650	**44.97**	10865, 10878
**24.71**	1870, 4124	**48.46**	ND
**25.00**	4124, 4212, 4908	**49.09**	ND
**25.18**	4211, 4124	**50.42**	ND
**25.84**	4041, 4212	**50.79**	ND
**26.54**	4795, 1953	**51.33**	ND
**26.94**	3989, 4007	**52.30**	15850, 15904
**27.17**	2088, 2867	**53.41**	28545, 28744
**27.58**	3341, 1671	**54.08**	25515
**28.24**	4084, 2091.5, 4717	**54.35**	24115
**28.47**	4786, 4085	**54.62**	ND
**28.65**	2372.5, 2851	**55.47**	ND
**29.04**	4745, 3504	**56.67**	ND
**29.28**	7234	**57.99**	ND

RT: Retention Time; ND: no determined; Ct28, Ct16, Ct17, Ct13, Ct1a, Ct6, Ct7, Ct25: clones from the cDNA library whose theoretical molecular mass coincides with the experimental mass.

### Transcriptome Analysis

As mentioned in the section of Material and Methods, the cDNA library produced about one million cfu/mL with 99% recombinant efficiency. From this library, 162 randomly chosen clones were sequenced, permitting to clearly identify 130 distinct clones. Some short sequences (32 in total) with low quality were discarded. The 130 good quality ESTs were clustered in 28 contigs formed by two or more ESTs each, and 49 singlets containing only one EST each. The mean nucleotide number (bp) of these ESTs was in the order of 330 pb (ranging from 116 to 635 pb), as shown in Figure S1 (A). The Length range of singlets (bp), which was very similar to the total ESTs, and the frequency of ESTs in the contigs are shown in Figure S1 (B and C, respectively).

### Nucleotide Similarity Search and Cell Function

**Figure 3:**
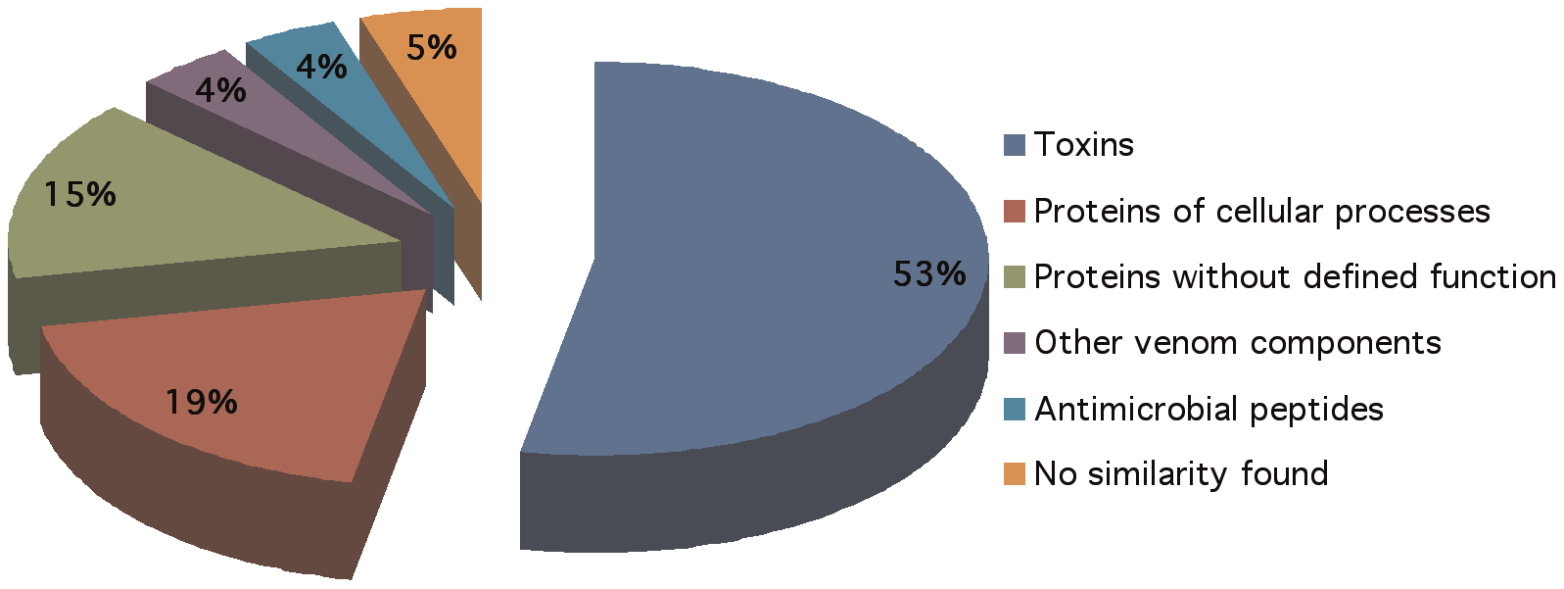
Transcripts from venom glands of *C. tecomanus.* Relative proportion (percentage) of each category obtained from the analysis of the transcripts from *C. tecomanus* venom gland library.

The ESTs sequences obtained were subjected to similarity search using the non redundant (nr) BLASTN and BLASTX databanks, taken an e-value of <10^−5^ as limit for homology confidence. Out of 130 ESTs, 53% corresponded to sequences that do contain similarities with scorpion toxin sequences (Figure 3), from which 81% (24 sequences) are similar to known toxins specific for Na^+^-channels and 19% (7 unique sequences) are similar to K^+^-channel specific toxins. From the remaining ESTs, 19% corresponds to proteins involved in cellular processes, 15% to hypothetical proteins without defined function, 5% with novel sequences without any similarity to other known proteins of the databases, 4% similar to metalloproteinases and other venom peptides; finally another 4% similar to antimicrobial peptides (Figure 3).

Concerning the putative functions of the peptides and proteins found among the ESTs, Table 2 gives a complete list, containing 70 amino acid sequences and their expected functions. The corresponding sequences are deposited in Genbank (EST database) under numbers JZ122265 to JZ122341.Thirty two of these sequences are identified as peptides, whose activity should be related to ion-channel recognition and function. It is worth mentioning that contig3 very likely corresponds to the same peptide earlier, described as toxin 1 (Ctl1) from *C.l.tecomanus* [7].

**Table 2:** Amino acid sequences deduced from ESTs of the cDNA library.

Na^+^-channel toxins								
Contig/Singlet		EST (ESTs)		ID/Genbank number		Descriptor		Amino acid sequence
Singlet		4608		JZ122266		Ct2, Putative Na^+^-channel toxin		MNSLLMITASLVLFGTVWT **KEGYLVNKSTGCTLVCVWLGKNNKCDMECKAKDQGGSYGYCKSNLCWCEGLPESTPTYPSYSKSCSSI**
Contig 3		4624, 5095, 5033, 4979		JZ122265		Ct1a, Putative Na^+^-channel toxin		MNSLLMITACLALIGTVWA **KEGYLVNHSTGCKYECFKLGDNDYCLRECRQQYGKGAGGYCYAFGCWCTHLYEQAVVWPLPKKTCNGK**
Contig 6		4607, 5084		JZ122279		Ct15, Putative Na^+^-channel toxin		MNSLLMITACLALVGTVWA **KEGYIVDYHTGCKYTCAKLGDNDYCLRECRLRYYQSAGGYCYAFACWCTHLYEQAVVWPLPNKRCKGK**
Singlet		5061		JZ122267		Ct3, Putative Na^+^-channel toxin		MNSLLMITACLALVGTVWA **KEGYIVDYHTGCKYTCAKLGDNDYCLRECRLRYYQSAGGYCYACACWCTHLYEQAVVWPLPNKRCKGK**
Singlet		5015		JZ122268		Ct4, Putative Na^+^-channel toxin		MNSMLMITACLALVGIVWA **KEGYLVNHHDGCKYACAKLGDDDCCLRECRAKYWKGAGGYCYAFACWCTHLYEQAVVWPLPNKTCYGK**
Contig 10		4611, 4824		JZ122283		Ct19, Putative Na^+^-channel toxin		MNSLLMITACLALVGIVWA **KEGYLVSHYDGCKYACAKLGDNDYCLRECKAKYWKGAGGYCYAFACWCTHLCEQAVVWPLPNKTCYGK**
Contig 9		4618, 4919, 5047		JZ122282		Ct18, Putative Na^+^-channel Alpha-toxin		MNSIFVVALTILLLGIARS **ELRDGYPLASNGCKFGCSGLGENNPTCNKICDEKAGSDYGYC<1?show=[sr]?>YWWTCYCQHVAEGTVLWGDSGTGPCMS**
Singlet		5098		JZ122269		Ct5, Putative Na^+^-channel toxin		MKIFIAFAFISALLLLAVES **ARDGYPVDEEGCKLSCFINDKWCNSACHYRGAKYGYCYTGGLACYCEAVPDNVKVWTYETNTCGKK**
Contig 13		4837, 4976, 4823		JZ122285		Ct21, Putative Na^+^-channel Alpha-toxin		MNSIFVVALTILLLGIERSES **RDGYPIASNGCKFGCSGLGENNPTCNHVCEKKAGSDYGYCYAWTCYCLHVAEGTVLWEDPGTGPCMT**
Contig 16		4925, 4949		JZ122286		Ct22, Putative Na^+^-channel Alpha-toxin		MKSIFVVALTILLLGIARS **ELRDGYPLASNGCKFGCSLGENNPTCNKICDEKAGSDYGYCYWWTCYCQHVAEGTVLWGDSGTGPCKS**
Singlet		4831		JZ122270		Ct6, Putative Na^+^-channel toxin		MKTFVLALCLVLIGMVYA **KDGYLVSKHTGCKLGCSPKIGDRYCHIECTSMNHKGDEGYCYWLACYCKGMPENAEVYPLPNKSCGK**
Contig 8		4827, 4609, 5070		JZ122281		Ct17, Putative Na^+^-channel Beta-toxin		MNSLLMITACLVLIGTVWA **KKDGYLVDKTGCKKTCYKLGENDFCNRECKWKHIGGSYGYCYGFGCYCEGMSDSTPTWPLPNKRCGKK**
Contig 11		4623, 4932, 5044		JZ122284		Ct20, Putative Na^+^-channel toxin		MNSLLMITACLVLFGTVWA **KDGYAVNKSTGCKIYCSVLGKYSFCDEECKAKHQGGSYGFCHSYACYCQGLPESTPTYPIPSKSCSRK**
Contig 20		4965, 5010, 5063		JZ122287		Ct23, Putative Na^+^-channel neurotoxin		MNSLLMITACLVLIGTVWA **KDGYAVNKSTGCKIYCSVLGKYSFCDEECKAKHQGGSYGFCHSYACYCQGLPESTPTYPIPSKSCSRK**
Contig 23		5042, 5034		JZ122288		Ct24, Putative Na^+^-channel neurotoxin		MNSLLMITACLLLIGTVWAKDGYAVNKSTGCKIYCSVLGKYSFCDKECKAKHQGGSYGFCHSYACYCQGLPESTPTYPIPSKSCSRK
Singlet		4935		JZ122271		Ct7, Putative Na^+^-channel toxin		MKVLILIIASVLLIGVEC **KDGYPMNSEGCKISCVIGNTFCDTECKMLKASSGYCWTLGLACYCEGLPENVEVWDSATNKCGGK**
Contig 24		5059, 4934		JZ122289		Ct25, Putative Na^+^-channel Beta-neurotoxin		MKVLILIIASVLLIGVEC **KDGYPKNSEGCKISCVIGNTFCDTECKMLKASSGYCWTLGLACYCEGLPENVEVWDSATNKCGGK**
Singlet		5013		JZ122272		Ct8, Putative Na^+^-channel toxin		MNYFILILVAALLIMDVNC **KKDGYPVDSESCRYNCWKNAYCDKICKEKKGESGYCYGWNLSCWCIGLPDNTNTKMNPFCQGLDGK**
Singlet		5088		JZ122273		Ct9, Putative Na^+^-channel toxin		MNYFILILVASLLILDVNC **KKDGYPIQENGCKYFCWKNDYCEKVCKDLKGEGGYCYANLSCWCIGLPDKVAIKHTQKCKRNGK**
Contig 2		5094, 4937, 5079		JZ122277		Ct13, Putative Na^+^-channel toxin		MKVLILIIASVLLIGVEC **KDGFPVDSEGCILLPCATRAYCSVNCKFMKGSGGSCDTLACHCKGLPEDAKVQDKPTNKCGRK**
Singlet		4946		JZ122274		Ct10, Putative Na^+^-channel toxin, partial		MDVALLFLITLSLVFIHNAES **KKEIPGGYPINRFECTYECAHADTDHIRCKNLCKKLGGSWGYCY**
Singlet		4954		JZ122275		Ct11, Putative Na^+^-channel toxin		MNVALLFLITLSLVLIHNA **ESKKNIPGGYPINENKCTYVCYHADKDKIRCSNFCKTLGASSGYCYWLTCYCEYLPDSVPQTNSIEVFSCGASIIGVNDNEYL**
Contig 5		5090, 4833, 4953, 5054, 5074		JZ122278		Ct14, Putative Na^+^-channel toxin -like peptide		MNVALLFLITLSLVLIHNAES **KKEIPGGYPINQFECTYECAHADTDHIRCKNLCKKLGGSWGYCYWNTCYCEYLPDSVPQKNSIEVFSCGATIIGVPDTE**
Contig 7		4616, 5055, 5067, 5083, 4928, 4929, 4968		JZ122280		Ct16, Putative Na^+^-channel neurotoxin		MNYFILLFVATFLLLDVNC **KKDGYPVDANNCKFECWKNEYCDELCKAKRAESGYCYKLKLSCWCEGLPDDEPTKTSDRCYGTGR**
Contig 25		5068, 4954		JZ122290		Ct26, Putative Na^+^-channel toxin		MNVALLFLITLSLVLIHNAES **KKNIPGGYPINENKCTYVCYHADKDKIRCSNFCKTLGASSGYCYWLTCYCEYLPDSVPQTNSIEVFSCGASIIGVNDNEYL**
**K^+^-channel toxins**								
**Contig/Singlet**		**EST (ESTs)**		**ID/Genbank number**		**Descriptor**		**Amino acid sequence**
Singlet	4961		JZ122291		Ct27, Putative K^+^-channel toxin		MKAFYGILIILLFCSMFNLNES **TIINVKCTSPKQCLLPCKQIYGPHVGAKCMNGKCHCSKIG**
Singlet	4973		JZ122292		Ct28, Putative K^+^-channel toxin		MKAFYGILIILLFCSMFKLNES **TTINVKCTSPKQCLKPCKDLYGPHAGAKCMNGKCKCYNNG**
Contig 14	5089, 5081		JZ122332		Ct33, Putative K^+^-channel toxin		MAGIAKITLILLFLFVTMHTFA **NWNAEAAVCVYRTCDKDCKRRGYRSGKCINNACKCYPYAK**
Contig 17	4960, 4920		JZ122293		Ct29, Putative K^+^-channel toxin		MKTFGFLLVLLLFSVMIATFSMVEA **YGCEGCKGKCSTRGKCINGRCKCYGRSDFFQEYEE**
Contig 18	4970, 5062, 4619		JZ122294		Ct30, Putative K^+^-channel toxin		MVAVGRRWIWALLASLLLLHSLAEA **GRGKEIMNKIKKKLADAKVTVKGAWDKLTSKSEFACPVIEKFCEDHCAAKESVGKCEDFKCLCLKPE**
Contig 27	4830, 4959		JZ122296		Ct32, Putative K^+^-channel toxin, partial		MVVHKATKSQFGCPLYEGYCETHCQDISNKDGDCHGMKCKCE
Contig 21	5016, 5082		JZ122295		Ct31, putative K^+^-channel toxin, partial		GAQSGCKALSCRGRFGIRPLKSRVVWECSPKQVVNST
**Proteins involved in cellular processes**								
**Contig/Singlet**		**EST (ESTs)**		**ID/Genbank number**		**Descriptor**		**Amino acid sequence**
Singlet		4613		JZ122333		Ct34, Putative translation elongation factor, partial		GEPIYLVEIQCPENAVGGIYGVLNRRRGHVFEECQVAGTPMFIVKAYLPVNESFGFTADLRSNTGGQAFPQCVFDHWQILPGDPMDGKSRPYQIVMDTRKRKGLKEALPDLDQYLDKL
Singlet		4614		JZ122297		Ct35, putative protein, partial		GSSLDLNRLGKTVPHFLGVPPAVPPRMSPSTATPKKFIGALGSEESQLIQDDNTDFADFTQFDESGTDLGANSQPQLKSGAFEIYKKPFTHTNSVPINASSGLTNFSDEILTSLSPTDDNTALLVEIEHPEDALMDRRRHVSAPPLALAATTITSVPRDKREIQSAIRAHRERNMMLSLLNSELNQELSEIMEERIALESAR
Singlet		4617		JZ122298		Ct35, Putative hemocyanin subunit 3b, partial		GEELERGDGITEDRTEYCSCGWPQHLLVPKGNAKGMVFYLFVMLTDRDHDKVVDGAGDHSICSDAVSYCGAKDDKYPDKKSMGFPFDRPLPFETVEEFLTSNMHIQEITVKFHP
Singlet		4826		JZ122299		Ct36, Putative copper transport protein, partial		AMASTHEFQVEMTCEGCSGAVKRVLDKLGDKINKVDIDLEKQRVYVESSMSSEELLVAIKKTGKTCSYVGVKS
Singlet		4834		JZ122300		Ct38, Putative protein, partial		IAMPFKPVEQAKCPKCGKSVYAAEEMLAAGQKWHKTCFKCGLCHKRLDSTNATEHGGELFCKQCYGRKFGPKGYGFGGGAGCLSMDKGEHLGNTDCVSNKPLDPNYN
Singlet		4922		JZ122301		Ct39, Putative protein, partial		LEEGFCRIKEKMASPLMMDIEMMNRYVSPVNPAVYPHLTLVLLGIGIFFMAWFFVYEVTSTKFTRDLFKELLISLVAAVFLGFGILFLLLWVGIYV
Singlet		4944		JZ122302		Ct40, Putative adaptin ear-binding coat-associated protein, partial		QDNMEYERVLLVKQEVFVYRIPPRSTSRGYRASDWKLDAPDWTGRLRVVAKDNDCILKLEDKNTGELFASCPVDKYPGVAIEAVLDSSRYFVLRIQDGNGRSAFIGIGFADRSDSFDLNVALQDHFKWLQKSEELENPVTDTTPPLDLSFKEGETITINMNITKKGGSARQRQNPAIRA
Singlet		4945		JZ122334		Ct41, Putative ribosomal protein 60 s, partial		GGKGSRSIVMTKGTSSFGKRRNKTHTLCRRCGRSSFHIQKSRCAQCGYPDRKMRHYNWSEKGKRRKTTGTGRLRHLRKVWRRFRHGFREGGQAKSMKRGAAATGSAK
Singlet		4956		JZ122335		Ct42, Putative kinase and microtubule interacting protein, partial		NKRKSAMSTERFDSFSIDEHKLKQIKESERKKTLELLEEMRNRLQQEKLSELERQKEILLKNFEAELQRILHQKEEEIKKSRAETKKIKEHLTLQVRDAERRALQISPVANEGFNRKLAIEVVELKAAKRRLEEALNDALESDKRKQTS
Singlet		4972		JZ122336		Ct43, CHK1 checkpoint-like protein, partial		GVTGNQGSIPEREPEKRLPHPRKAAGAQITHSRHGEVV
Singlet		5011		JZ122303		Ct45, Putative steroid membrane receptor, partial		GDDVEIKSESWISLIISEIIYSPINLTLLMICVILIYKLWPGKESSSQSSENTELPPLKKQDMTLEELKKYDGKNIDGRICVAVNGKIFDVTRGKRFYGPGGPYEAFAGHDASRGLALFSIDTIKDEYDDLSDLNTMEMESVREWEMQFTDKYTLIGRLLKPGEEPTDYSEDDEASPDQNQRGMEETQTS
Singlet		5021		JZ122304		Ct46, Putative anti-proliferation factor, partial		NMMSEIRTASRFLANLLRLSNRLDNDQLKLFCGQLEYLLEQHYREHWFPEHPCKGSGYRCLRINHKMDPIIAKAGFACGLEEASLRNLFPNELTLWVDPKEVSYRIGENGGICILYDANKLQQQQQYSDYENQENRNLSSSTNENINSP
Singlet		5035		JZ122305		Ct47, putative RNA-binding protein, partial		GWYTTYNGTVPHQYQYSYNISQPGYQFQYYYPYENGSVVSYRDFPEGSIYSYQYNSSYPTGMRYHKTWNTTYYMVNGVKYYYNITQPSGSYQYQYPSL
Singlet		5045		JZ122339		Ct49, putative transcription factor, partial		ISLATLPVHENSMPELSTAEVDKLDKKMKKTDEVSGSETDSESEDSAAEVEIPEGSGVKIQDQSQMAQAAGINEELVSKAKQSRSEKKARKVMSKLGLKQVQGVGRVTIRKSKN
Singlet		5046		JZ122340		Ct50, putative NADH dehydrogenase subunit 4, partial		LPKAHVEAPVAGSIILAAVLLKLGSYGILRISSIFYVSIKSLSPILIRVSLLGGILTRLICILQTDIKSLVAYSSVCHISLILRGALTFSC
Contig 15		4923, 4967		JZ122312		Ct58, putative 60S ribosomal protein, partial		MVQRKQKKALESINSRLALVMKSGKYVLGYKQTLKTLRQGKAKLIIIANNTPPL
Singlet		5051		JZ122306		Ct52, Putative ADP/ATP translocase, partial		GGGSAFFKGAFSNVLRGTGGAFVLVLYDEIKNFIF
Singlet		5058		JZ122307		Ct53, Putative elongation factor 1-beta, partial		GDDETNMQEMEKMVRSINCDGLTWGASKLVPLAFGIHKLQIVCIVEDLKVSIDWLQEEIQNFEDYVQSVDIAAFQKL
Singlet		5075		JZ122308		Ct54, PI-type proteinase, partial		GEPTEQGTSSEPTNIEEILEELKEEQKDKSTQYISEQPEQETASEPTNIQELIEELKREQEEESTQNIWEPTEQGTSSEPTNIEEILGELKEEQKDKSTQYISEQPEQETASEPTNIQELIEELKREQEEESTQNIWEPTEQGTSSEPTNIEEILEELKEDQKEKSTQYISEQPEQETASEPTNIRN
Singlet		5091		JZ122309		Ct55, Putative ubiquitin A, partial		TLSDYNIQKESTLHLVLRLRGGN
Singlet		4621		JZ122310		Ct56, Putative truncated large tumor antigen, partial		CRIRKNLPHLPLNLKHKMNAIVVVNLFIAAYNGYK
Contig 26		5053, 4927		JZ122314		Ct60, putative protein, partial		MLKVIIIITITIFVIHSSDATCVKCKIKDRHLTRTDNEESFRQHPGKENQDESRHHKKKENEQERIHRDLKKGNEEEQIHRDHKKGNEEERIHRDHKKGNEESRYASRKGQNQEESRHASRKGQNQEEGRQHPGKENRDDSRHVKRGTKSEESRQHPGKENRDDSRHVKRGQNQ
**Antimicrobial peptide**								
**Contig/Singlet**		**EST (ESTs)**		**ID/Genbank number**		**Descriptor**		**Amino acid sequence**
Singlet		5071		JZ122315		Ct61, putative antimicrobial peptide, partial		VVINESEA **FFGSLLSLGSKLLPSVFKLFQRKKERSIHKRDLEDLYDPYQRNLEMERFLKQLPMY**
Singlet		4620		JZ122276		Ct12, Putative antimicrobial peptide		MKGKTLLVVLLVALLIAEELNSF **KFGGFLKKMWKSKLAKKLRAKGREMIKDYANRVLEGPQEEAPPAERRR**
Contig 19		4963, 4881, 4966		JZ122313		Ct59, Putative antimicrobial peptide, venom anionic peptide		MVRKSLIVLLLVSVLVSTFLTTDA **PASFDDDFDALDDMNDLDLDDLLDLEPADLVLLDMWANMLENSDFEDDFE**
**Venom components**								
**Contig/Singlet**		**EST (ESTs)**		**ID/Genbank number**		**Descriptor**		**Amino acid sequence**
Singlet		5048		JZ122341		Ct51, putative venom metalloprotease, partial		TTMIYLAGIFLFAIASAIPSGRVDVVFPSVETSRSGVKTVKFRALNEEIELKLEPAGEILAKNFAIVNQQPNSIDVEDLKRRIYRDSNNGAALLIDEDGPVTIQGIVNSKFRIEPYESSRTVKDGQVPHKIVEVINDEKSFLNDAVMQIDV
Contig 12		4832, 4964		JZ122311		Ct57, putative metalloprotease, partial		GMGKYYCSHATGLAKDADLIMLLVTRELGELKKDGTVKTTLSGIAYVSGVCKQCKKVGVSLDDSDYNERVDTVAHETAHLLGSLHDGEKSSQGGPVGNPGAVDCLQTDGYIMGDRSVNEFKFSECSKKCVKYALSLPEAKCVYESCGF
Singlet		5041		JZ122338		Ct48, putative venom neuropeptide, partial		GGNFWKIETRSKMMFGIWILCGTAFFYCHVDAFMEYSNMVPGYNALIRRSQKEPWWKGRMFDNLGYNQAYLVKRNFDEIDNVGFNDFGPGTGRSWLPKRNLELASYNSRRLRL
Singlet		4980		JZ122337		Ct44, Putative venom metalloprotease-1, partial		TTMIYLASIFLFAIASAIPSGRVDVVFPSVETSRSGVKTVKFRALNEEIELKLEPAGE
**Proteins without defined function**								
**Contig/Singlet**		**EST (ESTs)**		**ID/Genbank number**		**Descriptor**		**Amino acid sequence**
Contig 28		4830-2, 5076		JZ122324		Ct70, putative unnamed protein product, partial		MQLLLLTCLLQLIMVTNKAIASQISQIKHFFHCILVVVCPNSSMYLIMSGS
Contig 4		5096, 4975, 5018, 5097, 5073, 4975, 4957, 5020, 4931, 5014, 5066		JZ122323		Ct69, putative hypothetical protein, partial		QICLRPRCWIRFPLFRKRNKKGVCSTHENLHDLSSNRREPGWFLS
qSinglet		4921		JZ122316		Ct62, putative hypothetical, protein, partial		STSRPLKDELASHTDDLAPEPTARAVNSAGQKFNYELFNCNNFNIRYWSWNYRGCWHQTCPPIVTR
Singlet		4943		JZ122317		Ct63, putative phosphomannomutase, partial		YFNETEQSRERFVVIYKFMERDSSTICLLDVDGTVTKPRQSITPTMEAFLQKLKDKVVIGLVGGSDISKIAEQMGGMDVIKKYEYVFAENGLVAYKNGELIAKESIASYLGEEKVQKLINFCLGYMSKLILPVKRGNFIEFRNGLINI
Singlet		4951		JZ122318		Ct64, putative hypothetical protein, partial		EPSTQSLCECSPFWSSLFSFCVCLSCKICLHSSFFFLNFFIYVCIFLTYFLVYVDLGLYFFYYRLSDQ
Singlet		4977		JZ122319		Ct65, putative hypothetical protein, partial		GGDICHGKYSRRTIIEFQCGSGEGKPMFQFESEDCTYYFIWSTSLACENKKHCIISNGSESYDLTPLSKSTYTVNDLTGKNDLYYLSVCDS
Singlet		4983		JZ122320		Ct66, putative hypothetical protein, partial		GDEEEEEDEPLDLTGGASRREDSDVPPILLPANCPYPPPAHLLLRKSAFIEDVWDGEPAIFSDDEDCNFLGFRITTNINNGECCQNEDQDQLPGNIDDDDQEDVPLAYLKQYVQKSSQS
Singlet		5049		JZ122321		Ct67, putative hypothetical protein, partial		GRRDFGKMAKAVSNLMAKAPVMLKGVVESSKPRFAVFMKYARVELAPPSPAEIPQVIQGFNKLIGNVKSGVWKTATVKTAWLNTLVGMEITFWFFLGECIGKRKLIGYEV
Singlet		5080		JZ122322		Ct68, putative hypothetical protein, partial		RMTSTSTQDLVGEPDRHTSHPHGYGTTDNSPPDENDRSGLSYRRRIIHLKEESTSYVDLTRGVLLLMFGHCIWTIILFFTLTTPLAMIIIGAIRIKECPL

Signal peptides are underlined; mature peptides are shown in bold; propeptides proposed are shown in italics.

**Figure 4:**
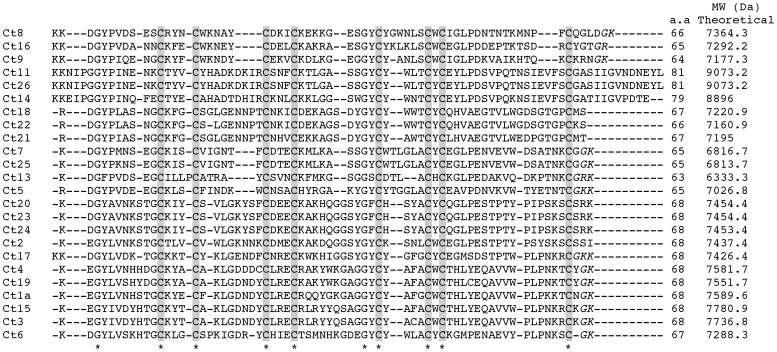
Multiple sequence alignment of putative Na^+^-channel toxin of *C. tecomanus.* Alignment of the 24 complete sequences of putative Na^+^-channel toxin of cDNA library from *C. tecomanus.* Amino acids identical in all sequences are indicated with an asterisk and the cysteines are shown shaded. The Ct11 and Ct26 sequences are identical at amino acid level but different at nucleotide level. The sequences of mature peptides Ct23 and Ct20 are identical, but the signal peptide of their respective precursors, are different. The amino acids that theoretically give rise to modification in the C-terminal (amidation) are shown in italics. The theoretical molecular weights were calculated taking into account these changes using the protparam program (http://web.expasy.org/protparam program).

**Figure 5:**
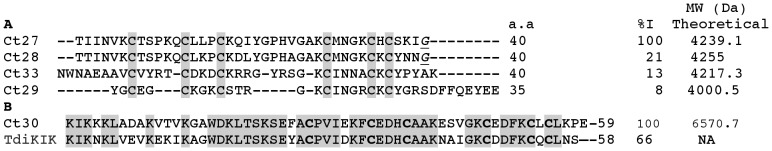
Sequence alignments of putative K^+^-channel toxins of *C. tecomanus*. **A**: sequence alignment of putative alpha-toxins found in the cDNA library of *C. tecomanus*, where the cysteines are shown shaded. The amino acids involved in a possible modification of the C-terminal are shown underlined (and in italics); the percentage of identity is indicated (% I). **B**: Sequence alignment of the putative β-toxin Ct30 of *C. tecomanus* with the sequence of TdiKIK toxin from *Tityus discrepans* (gb|ABE98264.1 |), which is the one showing the greatest identity. Identical amino acids are indicated in shade and are shown in bold. The number of amino acids (a.a) and the theoretical mass of each sequence of *C. tecomanus* are indicated. NA = Not applicable.

Figure 4 shows the deduced amino acid sequence of ESTs with important sequence similarities to other scorpion Na^+^-channel specific toxins reported in the literature. Twenty four complete sequences were identified showing approximately 68 residues (varying from 64 to 81) and theoretical molecular masses from 6813 to 9073 Da; ten residues are in corresponding positions in all sequences, including the 8 cysteines. The C-terminal sequence of fourteen sequences ends by the amino acid glycine followed by a basic residue (lysine) strongly supporting the suspicion that during maturation they are eliminated, providing the amine group for amidation of the previous residue [51]. Regarding K^+^-channel toxins, in the figure 5 shows in A the deduced amino acid sequence of four ESTs with sequence similarities to K^+^ channel α-toxins and their theoretical molecular masses and B shows the alignment of the deduced amino acid sequence of EST Ct30 compared with the K^+^ channel β-toxin TdiKIK.

### Amino Acid Sequencing of Venom Components by Edman Degradation

Based on the results of nucleotide similarity search and knowing the molecular masses of the components directly isolated from the venom, it was found that eight theoretical masses of clones sequenced coincided with 8 masses obtained by mass spectrometry determination (see table 1 and Fig. 4). The several fractions that coincided, obtained from the HPLC, were loaded into an automatic amino acid sequencer and their sequences were determined by Edman degradation. Table S1, shows the amino acid sequence of eight peptides, whose presence and primary structure were determined. The amino acid sequence deduced from the cDNA of these peptides were aligned in Fig.4, and confirmed by direct sequencing (see Table S1).

## Discussion

### Mass Spectrometry Analysis and HPLC Separation

The mass spectrometry analysis reported here shows that most peptidic components of *C. tecomanus* venom have a molecular weight above 4000 Da (Fig.2). Twenty peptides (19% of total) have molecular masses between 3500 and 4500 Da. As already mentioned, within this range of masses, are the peptides assumed to be K^+^-channel specific; whereas thirty components (29% of total) have molecular masses from 6000 to 7500 Da, corresponding to typical molecular weights of toxins that recognize Na^+^-channels [11]. An additional observation supporting these findings is the results of HPLC separation of scorpion venom of the family Buthidae. Batista et al., [28] showed that components eluting at retention time (RT) from 20 to 31 min from the same C_18_ reverse phase column, run in similar conditions, have molecular weights around 3000–5000 Da and correspond to K^+^-channel specific peptides. Components eluting from 30 to 40 min have molecular masses between 6000 to 8000 Da and are known to contain Na^+^-channel specific peptides. Similar results are shown in our Fig.1. In addition, components eluting at RT around 40 min and over, show molecular masses of 10,000–11,000 Da, for which their functions have not been completely identified yet [52]. Several of these venom components are species specific [11], affecting either invertebrates (insects, crustaceans), or vertebrates (mammalians, chickens). From the venom of related scorpions of the genus *Centruroides* several peptides highly toxic to mammals were isolated and characterized, for example: Cn2, Cn3 and Cn4 from *C. noxius* [53], [54], a toxin from *C. infamatus infamatus* [55] and seven toxins (Css1-7) from *C. suffusus suffusus* [56].

From the scorpion *C. tecomanus* five peptides toxic to mammalians were isolated and partially characterized [6]–[8]. Only toxin Clt1 was completely sequenced and shown to contain 66 amino acid residues, with 86% sequence identity to toxin II from *C. suffusus suffusus* toxin II [56]. Clt1 affects inactivation of Na^+^-channels, when assayed on dorsal root ganglion cells of chicken [7]. It is clear that the amino acid sequences found by cDNA cloning genes of venom glands of this scorpion (Table 2) and the mass spectrometry data (Table 1), together with the results of direct amino acid sequence determination (Table S1) of venom components isolated by HPLC (Fig.1), allowed to confirm the data obtained by other authors with scorpion venom of the genus *Centruroides.* The *C. tecomanus* venom is certainly a complex mixture of components (80 sub-fractions) containing toxins that certainly could be affecting K^+^- and Na^+^-channels, as demonstrated in other scorpion venoms [28], [52], [57], [58].

The correlation of the transcriptome with the proteome data analysis is relatively restrict due to two reasons, Most proteins of higher molecular weight present in the cDNA library of the venom glands are not secreted as part of the venom. The second reason concerns the information available, since some of the sequences obtained are still incomplete and the exact positions of signal peptide, mature peptide, propeptide and/or possible postraductional modifications are not known, hence it does not permit predicting the exact molecular mass of the expected protein or peptide. This makes difficult to correlate the sequences found with the proteome analysis. The precursor sequences from scorpion venom best identified are those from toxins that affect the function of ion-channels. The theoretical molecular mass expected for toxins, given the fact that sufficient information about their processing is known, allowed finding possible matching masses from the proteome analysis. The peptides with coincident masses where then selected from the HPLC separation of the venom and use for direct amino acid sequence determination, by automatic Edman degradation. This confirmed perfect coincidence of the molecular mass experimentally determined with that predicted from the cDNA cloning. Thus, it was possible to identify several putative toxins that in fact are present in the venom.

### Transcriptome Analyses

Previous work conducted with cDNA libraries obtained from venom glands of scorpions showed the possibility of identifying by the EST methodology, the presence of many genes coding for similar peptides as those directly identified from the scorpion venoms. The results obtained are encouraging, however more than 98% of the scorpion species existent in the world remain unknown (reviewed in [59]).

The first such analysis was conducted with a cDNA library from the venom glands of the Mexican scorpion *Hadrurus gertschi*, which belong to the family Caraboctonidae. Most known scorpion species containing toxic peptides to mammals belong to the Buthidae family. This pioneer work, conducted with a non Buthidae species, identified 147 ESTs of good quality, providing significant information about the kind of molecules present in the venom glands of this species [31]. Up until now 14 reports of cDNA libraries have been screened for their ESTs, from which about half of them are from Buthidae scorpions [32], [34], [36], [38]–[40], [43] and the rest are from non Buthidae species [31], [33], [35], [37], [41]–[44]. The results reported in this literature are highly variable both in terms of the number of sequences obtained and the putative functions attributed to the innumerous genes identified. In some cases, a great number of components were identified, like those obtained from *Scorpiops jendeki* (a total of 871 ESTs), where half of the proteins and peptides are part of venom itself and the other half is assumed to be components involved in cellular processes or belonging to hypothetical proteins of unknown function [43]. Another example is the global transcriptome analysis of the scorpion *Centruroides noxius* by means of the platform of massive pyrosequencing, using genes obtained from the cDNAs of venom glands, as well as cDNAs from the entire body of this species. In the order of three million readings were obtained and assembled, from which 72 isogroups containing peptides similar to toxins previously reported for other scorpions were identified [39]. However, other examples are available using a more restrict number of different genes identified, for instance 118 ESTs were reported from the non Buthidae scorpion *Opisthocanthus cayaporum* [33].

In the present report dealing with ESTs of *C. tecomanus* the percentage of putative toxins found (53%) is higher than other proteins and peptides, such as those involved in cellular processes, which are only 19%. This could explain the high toxicity of the venom from *C. tecomanus;* one of the most dangerous scorpions [5]. It is worth mentioning that some discrepancies can occur due to uncontrolled external factors, such as stress and environmental conditions, as pointed out by [36], [60].

Since from *C. tecomanus* the information available is restricted to five toxic peptides, as mentioned earlier [6], [7], the transcriptome analysis reported here contains a substantial contribution to advance the knowledge on venom and venom gland components of this highly toxic scorpion. Novel information concerning the presence of toxic peptides similar to Na^+^-channel and K^+^-channel specific peptides, similar to antimicrobial peptides and the presence of proteolytic enzymes complete the identification of molecules into this venom, as it will be further discussed below. Among the advantages of the present transcriptome analysis is that by characterizing the nucleotide sequences of the ESTs isolated we have been able to identify toxic peptides, their precursors and maturation processes, as well as allowed identifying proteins related to structural and metabolic processes taking place in the venom glands, information which would be almost impossible to obtain only by classical biochemical characterization of venom components.

Contig3 (Ct1a of Table 2) very likely corresponds to toxin Clt1 previously described, with small corrections: position 62 of contig3 is lysine and position 66 is asparagine (see figure S2-letter C). The absence of serine and presence of asparagine at position 66 is also conserved in toxins of the published sequence of Cll2 from the scorpion *C. limpidus limpidus*, and toxin Cii1 of *C. infamatus infamatus* (Fig. S2-letter C). Several similar Na^+^-channel specific peptides were found, as shortly described below.

### Toxins Specific for Na+-channels

Peptides with amino acid sequence similar to toxins that recognize Na^+^-channels were found to be quite abundant. Twenty four sequences correspond to similar known toxins (Fig.4), from which seven were confirmed by Edman degradation (Table S1). It is worth mentioning that two sequences are listed twice in Fig.4 (Ct11 and Ct26; Ct20 and Ct23), because their differences were found in the nucleotide sequence or signal peptide sequence, but the mature segment contains the same sequence. It is well described in the literature that these peptides are modulators of Na^+^-channel activity [15], [16], [61]. These peptides are commonly found in scorpions of the family Buthidae [32], [34], [62], and are responsible for intoxication with serious medical problems, because they affect Na^+^-channels of excitable tissue causing membrane depolarization, liberation of neurotransmitters, which then affect the proper functioning of several organs that might lead to respiratory distress or heart failure, the two most common cause of dead [63], [64]. These peptides contain between 58 to 76 amino acid residues, tightly stabilized by four disulflide bridges [11]. Amino acid sequence comparison of peptides listed in Fig.4, Table 2, Table S1 and Fig.S2 showed high degree of similarity among themselves and with other known peptides purified from scorpion venom, as it can be seeing in the following few examples. Peptides Ct7 (singlet 4935) and Ct25 (contig24) have only one amino acid different in their primary structure and show 60 and 62% identity with RjAa8 from the scorpion *Rhopalurus junceus.* Ct20 (contig 11) showed 74% identity to toxin Cex3 of *Centruroides exilicauda* (Fig. S2-letter B). Ct15 (contig 6) and Ct4 (singlet 5015) have 84% identity to toxin Cll3 from *C. limpidus limpidus* (Fig. S2-letter A). Peptides Ct18 (contig 9), Ct22 (contig 16) and Ct21 (contig 13) showed 90, 88 and 91% identity to Cn12 from the scorpion *C. noxius*, which is structurally similar to the β-ScTX, but has an α-ScTx effect [65].

### Toxins Specific for K+-channels

Nineteen percent of the sequences found showed similarities to toxins specific for K^+^-channels. Seven unique sequences were identified (2 singlets and 5 contigs, see Table 2). Peptide Ct28 (Table S1) is the only one, whose primary structure was directly determined by automatic sequencing. Among these peptides are those of α- as β-K^+^-channel types, showing higher similarities to the known toxins isolated from scorpions of the genera *Centruroides* and *Tityus.* Most of these peptides are blockers of K^+^-channels; containing 23 amino acid residues (the α-type) to 64 residues (β-type), very well packed by three to four disulfide bridges [13]. Ct27, Ct28, Ct29 and Ct33 (Table 2) showed similar sequences, containing from 35 to 40 amino acids, stabilized by 3 disulfide bridges (Fig. 5A) and are very likely α-K^+^-channel specific toxins, whereas Ct30 (Table 2) contains 59 amino acid residues, stabilized by three disulfide bridges, whose sequence is 66% identical to toxin TdiKIK of the scorpion *Tityus discrepans* (Fig.5B), a β-K^+^-channel peptide.

### Antimicrobial Peptides (AMPs)

The AMPs peptides were found in some scorpion transcriptome studies [31], [35], [37], [38]. These peptides play an important role in the innate immune system, because they can depolarize neuronal cells and induce immobilization of preys but also can potentiate the effect of other neurotoxins [66]. Additionally, they constitute the first line defense against infection by pathogenes. The AMPs are short chain cationic and anionic peptides [67], normally divided into various groups according to their primary and secondary structures. The most extensively studied are lineal amphypatic peptides without disulfide bridges that can form α-helices [41], also known by the abbreviation NDBP, meaning non-disulfide-bridge peptide. Another group is composed by peptides rich in cysteines that form one or more disulfide bridges [68]. Finally, there are specific peptides, rich in certain amino acids such as glycine, proline and histidine [69].

In this communication we report the presence of three putative PAMs: Ct12 (singlet 4620, Ct61 (singlet 5071 and Ct59 (contig 19), as shown in Table 2. The sequence 4620 corresponds to a peptide similar to the antimicrobial peptide MeVAMP-1 isolated from the scorpion *Mesobuthus eupeus,* showing 74% similarity. This peptide could be classified as NDBP or as a specific peptide, because it contains high percentage of glycine and proline. The sequence 5071 codes for a precursor (still lacking the N-terminal segment, where the signal peptide is missing), whose segment coding for the mature peptide is complete and it belongs to the NDBP-5 class, and its sequence is 65% similar to the antimicrobial ponericin-W-like 32.1 peptide of the scorpion *Lychas mucronatus*. Finally, the contig 19 sequence is similar to anionic peptides of the family NDBP-6.2, showing 70% identity to the acidic peptide Ka2 from the *Mesobuthus martensii*. The putative AMPs described here constitute original information, not known thus far to exist in this scorpion venom. It is clear that in due time these peptides need to be either isolated from the venom or chemically synthesize and their real function determined.

### Metalloproteases

Additional and important components found in scorpion venoms are enzymes with proteolytic activity [70], [71]. Two main types of proteases are described: serineproteases (SPSVs) and metalloproteases [37], [38], [72].

Here we identified three amino acid sequences corresponding to three different putative metalloproteases (see Table 2). Ct57 (contig 12) shows 56% similarity to antarease, a metalloproteinase found in the venom of the scorpion *Tityus serrulatus*, described to be responsible for pancreatitis of individuals stung by this species of scorpion. Antarease cleaves the vesicle-associated membrane protein 2: VAMP2 and VAMP8. These proteins are associated with zymogen granule membranes in pancreatic acinar cells [46].

Two additional sequences coding for putative metalloproteinase are Ct44 (singlet 4980) and Ct51 (singlet 5048), which present respectively 52% and 47% similarity to MeVMEP-1 (Genbank number ABR20110.1), a Zinc dependent metalloprotease isolated from de scorpion *Mesobuthus eupeus*.

### ESTs Sequences of Proteins Involved in Cellular Processes and Other ESTs of Proteins without Defined Function

Nineteen percent of the ESTs sequences obtained correspond to proteins involved in various cellular processes (structural, metabolic, transport, biologic processes, mitochondrial genes and others, see Table 2). However 15% of sequences found correspond to proteins without defined function (hypothetical proteins), and 5% of ESTs do not match with any sequence into the databases. They could be specific to scorpions, not reported for other organisms. Thus, again this is original information not known previously. Since the information is deposited in databank, it will certainly help future identification of similar components in other scorpion venom samples, although the function associated to these proteins still remain to be determined.

### Conclusions

This communication reports the identification of representative examples of each one of the proteins and peptides known to be present in scorpion venom and scorpion venom glands. An important number of the cloned genes code for peptides thought to be toxic to Na^+^- and K^+^-ion-channels, confirming the experimental facts that stings by this species of scorpion could be dangerous to humans. The knowledge of the structure of these peptides certainly will help developing strategies for using this information for designing better or novel anti-venoms. Biochemical, proteomic and transcriptomic characterization of venom components from *C. tecomanus,* as reported here, are important and necessary for identification of biological functions of these components, but also provide information that eventually can be used for development of new pharmaceutical usefull drugs, such as immunomodulators, specific ion-channel blocker and antibiotics.

## Supporting Information

Figure S1 **Distribution of sequence lengths of ESTs, singlets and contigs of** ***C. tecomanus*** **. A)** A total of 130 ESTs were analyzed in the transcriptome of *C. tecomanus*. The X-coordinate is the length of sequences in 50 bp intervals, whereas the total number of ESTs for each cluster is shown in the Y-coordinate. **B)** This panel shows the length range distribution of singlets (bp) indentified in the cDNA library from *C. tecomanus* **.** A total of 49 singlets were obtained in the transcriptome of *C. tecomanus*. **C)** This panel shows the ESTs distribution in the contigs of the cDNA library from *C. tecomanus*. A total of 28 contigs were obtained.
(TIF)

Figure S2 **Multiple sequence alignment of Na^+^-channel toxins of** ***C. tecomanus***. **A**: Alignment of sequences from **Ct15** and **Ct4** regarding Cll3 toxin from *Centruroides limpidus limpidus* (GenBank: AAP49502.1). **B**: sequence alignment of **Ct20** and Cex3 toxin from *Centruroides exilicauda* (GenBank: AAT97994.1). **C**: Sequence alignment of the sequence **Ct1a**, of this study, compared with Clt1 toxin from *Centruroides tecomanus* (UniProtKB/Swiss-Prot: P18926.1), Cll2 toxin from *Centruroides limpidus limpidus* (UniProtKB/Swiss-Prot: P59898.1) and Cii1 toxin from *Centruroides infamatus infamatus* (UniProtKB/Swiss- Prot: P59897.1); different amino acids between the sequences are shown in bold. The percentage of identity (%) of each alignment is indicated and the cysteines are shown shaded in gray.
(TIF)

Table S1 **Complete amino acid sequence of C. tecomanus peptides as found by Edman degradation and genes cloned from cDNA library.**
(DOC)
