# Supplementary figures and images for: Mass Fingerprinting of the Venom and Transcriptome of Venom Gland of Scorpion Centruroides tecomanus
Source: PLoS One. 2013 Jun 20;8(6):e66486. doi: 10.1371/journal.pone.0066486 (PMC3688770; doi:10.1371/journal.pone.0066486)

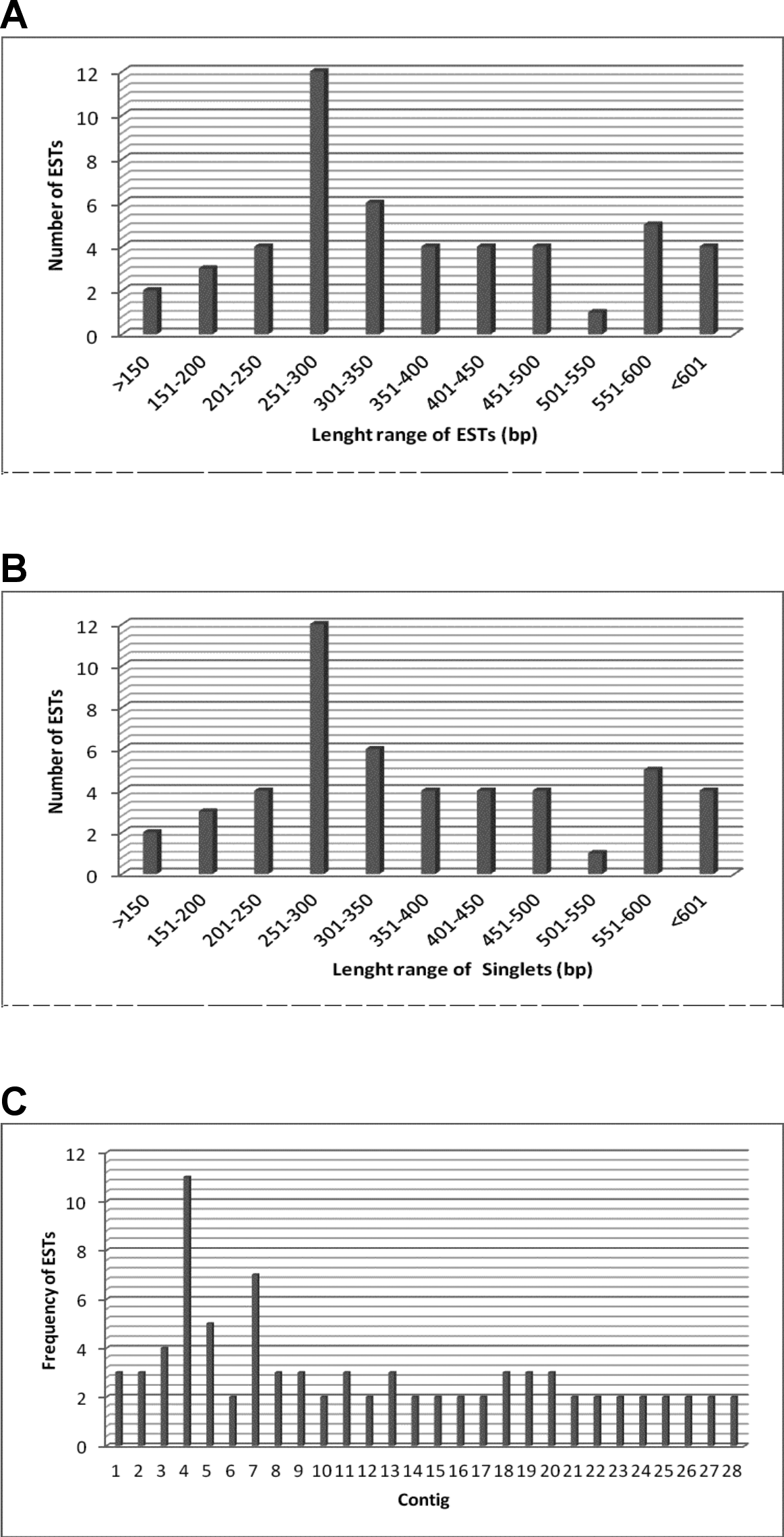

Supplement: Figure S1 — Distribution of sequence lengths of ESTs, singlets and contigs of C. tecomanus . A) A total of 130 ESTs were analyzed in the transcriptome of C. tecomanus. The X-coordinate is the length of sequences in 50 bp intervals, whereas the total number of ESTs for each cluster is shown in the Y-coordinate. B) This panel shows the length range distribution of singlets (bp) indentified in the cDNA library from C. tecomanus . A total of 49 singlets were obtained in the transcriptome of C. tecomanus. C) This panel shows the ESTs distribution in the contigs of the cDNA library from C. tecomanus. A total of 28 contigs were obtained. (TIF) [file pone.0066486.s001.tif]

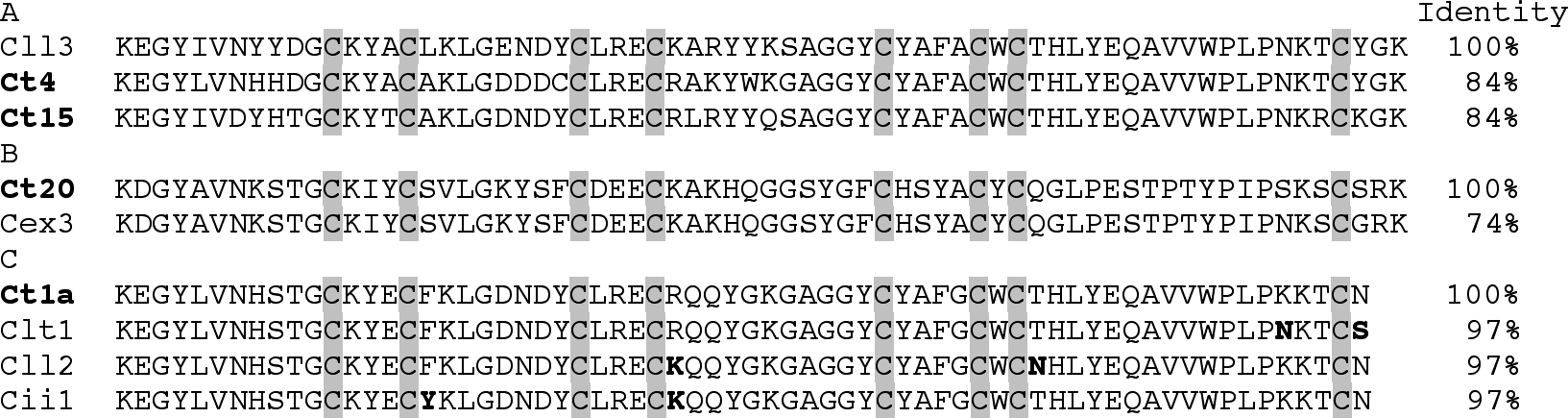

Supplement: Figure S2 — Multiple sequence alignment of Na+-channel toxins of C. tecomanus. A: Alignment of sequences from Ct15 and Ct4 regarding Cll3 toxin from Centruroides limpidus limpidus (GenBank: AAP49502.1). B: sequence alignment of Ct20 and Cex3 toxin from Centruroides exilicauda (GenBank: AAT97994.1). C: Sequence alignment of the sequence Ct1a, of this study, compared with Clt1 toxin from Centruroides tecomanus (UniProtKB/Swiss-Prot: P18926.1), Cll2 toxin from Centruroides limpidus limpidus (UniProtKB/Swiss-Prot: P59898.1) and Cii1 toxin from Centruroides infamatus infamatus (UniProtKB/Swiss- Prot: P59897.1); different amino acids between the sequences are shown in bold. The percentage of identity (%) of each alignment is indicated and the cysteines are shown shaded in gray. (TIF) [file pone.0066486.s002.tif]
