# Supplementary material for: Mass Fingerprinting of the Venom and Transcriptome of Venom Gland of Scorpion Centruroides tecomanus
Source: PLoS One. 2013 Jun 20;8(6):e66486. doi: 10.1371/journal.pone.0066486 (PMC3688770; doi:10.1371/journal.pone.0066486)
Supplement: Table S1 — Complete amino acid sequence of C. tecomanus peptides as found by Edman degradation and genes cloned from cDNA library. (DOC) [file pone.0066486.s003.doc]

**Table S1:** Complete amino acid sequence of *C. tecomanus* peptides as found by Edman degradation and genes cloned from cDNA library

| RT | cDNA Library clone | SEQUENCE | MW Expected (Da) | MW Determined(Da) |
| --- | --- | --- | --- | --- |
| 21.14 | Ct28 | **TTINVKCTSPKQCLKPCKDLYGPH**AGAKCMNGKCKCYNN* | 4255 | 4255 |
| 30.92 31.21 | Ct16 | **KKDGYPVDANNCKFECWKNEYCDELCKA**KRAESGYCYKLKLSCWCEGLPDDEPTKTSDRCYGT* | 7292.2 | 7292 |
| 32.93 | Ct17 | **KKDGYLVDKTGCKKTCYKLGEN***D***FCNRECK**WKHIGGSYGYCYGFGCYCEGMSDSTPTWPLPNKRC* | 7426.4 | 7427 |
| 34.05 34.26 | Ct13 | **KDGFPVDSEGCILLPCATRAY***C***SVN**CKFMKGSGGSCDTLACHCKGLPEDAKVQDKPTNKC* | 6333.3 | 6333 |
| 35.19 35.92 | Ct1a | **KEGYLVNHSTGCKYECFKLGDNDYCL**RECRQQYGKGAGGYCYAFGCWCTHLYEQAVVWPLPKKTCN* | 7589.6 | 7591 |
| 38.65 | Ct7 | **KDGYPMNSEGCKISCVIGNTFCDTECKM**LKASSGYCWTLGLACYCEGLPENVEVWDSATNKCG* | 6816.7 | 6817 |
| 37.03 | Ct6 | **KDGYLVS***KH***TGCK**LGCSPKIGDRYCHIECTSMNHKGDEGYCYWLACYCKGMPENAEVYPLPNKSC* | 7288.3 | 7288 |
| 39.61 | Ct25 | **KDGYP***K***NSEGC***K***I***S***C***V***I**GNTFCDTECKMLKASSGYCWTLGLACYCEGLPENVEVWDSATNKCG* | 6813.7 | 6814 |

RT means retention time. The bold letters were amino acids identified by Edman degradation (a few residues in italics were not directly determined), whereas normal letters were obtained from cDNA analysis. Asterisks mean C-terminal amidated. Cl28 is the only K^+^-channel specific peptides, the others are typically Na^+^-channel specific peptides.
